# Contrasting effects of sleep fragmentation and angiotensin-II treatment upon pro-inflammatory responses of mice

**DOI:** 10.1038/s41598-022-19166-9

**Published:** 2022-08-30

**Authors:** David C. Ensminger, Nicholas D. Wheeler, Reem Al Makki, Kristen N. Eads, Noah T. Ashley

**Affiliations:** 1 Department of Biology, Western Kentucky University, Bowling Green, KY USA; 2 Department of Biological Sciences, San José State University, San Jose, CA USA; 3 College of Veterinary Medicine, Mississippi State University, Starkville, MS USA; 4 School of Physician Assistant Studies, Lipscomb University, Nashville, TN USA

**Keywords:** Cardiovascular biology, Cytokines, Inflammation

## Abstract

Disordered sleep promotes inflammation in brain and peripheral tissues, but the mechanisms that regulate these responses are poorly understood. One hypothesis is that activation of the sympathetic nervous system (SNS) from sleep loss elevates blood pressure to promote vascular sheer stress leading to inflammation. As catecholamines produced from SNS activation can directly regulate inflammation, we pharmacologically altered blood pressure using an alternative approach-manipulation of the renin-angiotensin system (RAS). Male C57BL6/J mice were treated with angiotensin or captopril to elevate and reduce blood pressure, respectively and then exposed to 24-h of sleep fragmentation (SF) or allowed to sleep (control). Pro- and anti-inflammatory cytokine gene expression and as endothelial adhesion gene expression as well as serum glucocorticoids (corticosterone) were measured. RAS manipulation elevated cytokines and endothelial adhesion expression in heart and aorta while SF increased cytokine expression in peripheral tissues, but not brain. However, there were interactive effects of angiotensin-II and SF upon cytokine gene expression in hippocampus and hypothalamus, but not prefrontal cortex. SF, but not RAS manipulation, elevated serum corticosterone concentration. These findings highlight the contrasting effects of RAS manipulation and SF, implying that inflammation from SF is acting on different pathways that are largely independent of RAS manipulation.

## Introduction

It is well appreciated that “a good night’s sleep” is beneficial and can lead to recovery from critical illness and injury^1^, elevated pain tolerance^2^, and improved cognition^3^. However, disrupted or fragmented sleep exposes individuals to a myriad of negative health effects such as reduced antioxidant concentrations^4^, diminished immune function^5^, decreased neurogenesis^6^, and poor mental health^7^. Mechanistically, there is accumulating evidence that sleep loss activates a pro-inflammatory phenotype in brain and peripheral tissues^5,8^, which can putatively lead to chronic pathologies such as cardiovascular and metabolic diseases. As such, these diseases are manifested in a number of sleep disorders that include insomnia, shift work, and obstructive sleep apnea^9^.

Typically, inflammation is a non-specific reaction to injury, infection, or pollutants that functions to protect the body and then promote healing^10,11^. However, the mechanisms for induction and maintenance of inflammation specifically arising from sleep dysfunction remain unclear. Disrupted sleep alters neuroendocrinological stress responses, such as activation of the hypothalamic–pituitary–adrenal (HPA) axis^12^ and sympathetic nervous system (SNS)^13^, leading to the release of glucocorticoids and catecholamines, respectively, from the adrenals. It has been proposed that alterations in these immunoregulatory hormones may contribute to this pro-inflammatory phenotype^14^. This hypothesis has received empirical support using chemical sympathectomy and pharmacological blockade of adrenergic receptors to demonstrate that SNS suppression ameliorates inflammation from experimental sleep fragmentation in mice^15,16^. An additional hypothesis to explain inflammation from sleep loss involves vascular changes associated with increased blood pressure as a result of increased sympathetic output from prolonged wakefulness^14,17^. Typically, blood pressure drops to its nadir during normal, healthy sleep and endothelial markers also decrease^18^. However, during experimental sleep deprivation, blood pressure and other indicators of sympathetic output increase as well as pro-coagulatory markers induced by activated vascular endothelium, such as E-selection and I-CAM^19^. It has been proposed that vascular sheer stress associated with increased blood pressure activates microvasculature and leads to production of inflammatory mediators during sleep loss^14,20^.

To examine the vascular stress hypothesis, it was necessary to modulate blood pressure to assess inflammatory responses independent from SNS control because previous studies have reported direct effects of catecholamines upon inflammatory responses to sleep fragmentation^21^. Therefore, we decided to modulate blood pressure independent of the SNS through altering the renin-angiotensin system (RAS). Upon RAS stimulation, angiotensinogen is cleaved by renin and ACE to form angiotensin II (Ang II), which promotes hypertensive effects by acting on type 1 and 2 G-protein coupled receptors. It should be noted that Ang II treatment can also have direct pro- and anti-inflammatory effects depending upon target receptor type present^22,23^. Nonetheless, we utilized this alternative method as a means to broadly compare inflammatory responses from two stressors that tend to promote hypertension: sleep loss and angiotensin-II treatment.

The aims of this study were to compare and contrast inflammatory responses to RAS manipulation, sleep fragmentation, and their interaction in male mice to help elucidate the role that elevated blood pressure may play in mediating inflammation from sleep loss. We used exogenous Ang II administration and captopril (Cap) to increase and decrease blood pressure, respectively. Cap inhibits Ang II production via inhibition of ACE and is commonly prescribed for the treatment of arterial hypertension^24^. If inflammatory responses are similar between mice exposed to angiotensin versus mice subjected to sleep fragmentation, then this would suggest a potential role for increased blood pressure in mediating inflammatory responses. Alternatively, if these two groups diverge in inflammatory responses, then this result would imply that other mechanisms (e.g., direct effects from catecholamines, glucocorticoids) are responsible for promoting inflammation from sleep fragmentation. Additionally, we predicted that exogenous Ang II treatment would compound the inflammatory response to sleep fragmentation and increase activation of adhesion markers such as E-selectin and ICAM-1. Further, we predicted that Cap treatment would lead to decreased inflammatory responses to sleep fragmentation as well as reduced activation of endothelial markers. Finally, we predicted that sleep fragmentation would elevate glucocorticoids (CORT) and that RAS manipulation would have no effect on HPA activation.

## Methods

### Animals

Male adult C57L/6 J mice (20–25 g; *n* = 60; Jackson Laboratory, Bar Harbor, ME) were housed under standard rodent colony conditions (lights on: 0800–2000 h, 21 °C ± 1 °C) at Western Kentucky University and given food and water ad libitum. Mice were group housed post-weaning (21 days of age) in polypropylene cages with littermates and provided with corncob bedding and enrichment. All procedures were approved by Western Kentucky University’s Institutional Animal Care and Use Committee (#19-14) and followed the National Institutes of Health’s “Guide for the Care and Use of Laboratory Animals.” Reporting in the manuscript follows the recommendations in the ARRIVE guidelines.

### Study design

Male mice (8–9 weeks of age) were placed in an automated sleep fragmentation chamber (model 80390; Lafayette Instrument Company, Lafayette, IN) in groups ≤ 5 mice with corncob bedding and enrichment. Food and water were provided ad libitum. Mice were tagged with numbered ear tags for individual identification and allowed to acclimate to the new cage setting for 6 days prior to the start of the study^25^. This sleep fragmentation chamber has been shown to effectively reduce sleep in mice as measured by telemetric transmitters to record electroencephalogram (EEG) in brain and electromyogram (EMG) in nuchal muscle^26–28^.

To assess the effect of elevated or reduced blood pressure on inflammatory and adhesion markers due to sleep fragmentation (SF), Ang (vasoconstrictor, n = 19) and Cap (ACE inhibitor; n = 20) were used to raise and lower blood pressure, respectfully, compared to control. Ang and Cap were dissolved in 0.9% NaCL and administered via micro-osmotic pumps (0.25uL/hr; Alzet model 1002, Durect Corporation, Cupertino, CA 95014) to achieve ~ 800 ng/kg/min for Ang and ~ 3 mg/kg/day for Cap; these doses have been shown previously to elevate and reduce blood pressure, respectively^29,30^. Saline was added to the micro-osmotic pumps of control animals as a vehicle control (Con; n = 21). Pumps were implanted using aseptic surgery techniques and animals were monitored for recovery. Treatments were randomized using a random number generator for each animal. The impacts of Ang, Cap, and Con on blood pressure were validated using DSI telemetry in a subset of mice (PA-C10; Data Science International, St. Paul, MN 55112). As expected, Ang increased blood pressure and Cap decreased blood pressure compared to Con following 8 days of infusion (Supplementary Fig. 1).

### Sleep fragmentation and sample collection

After 8 days of drug treatment, half of each drug treatment group experienced SF or no SF in a full factorial design (NSF; NSF-Ang *n* = 10, NSF-Con *n* = 11, NSF-Cap *n* = 10, SF-Ang *n* = 9, SF-Con *n* = 10, SF-Cap *n* = 10). Acute SF was induced via a sweeping bar set to move horizontally every 120 s for 24 h (starting at lights on: 0800) across the cage and has been validated with non-invasive real-time monitoring of sleep using piezoelectric technology^15^. This rate of SF was chosen as it approximates the rate of arousals observed in patients with severe sleep apnea^31^. For the NSF group, the bar remained stationary. At the end of the 24 h of SF treatment (day 9), all mice were euthanized via rapid isoflurane induction (< 1 min) followed by decapitation. The euthanizer and sample collector were blind to the treatments.

Trunk blood was collected < 3 min of initial handling of mice, stored on ice for < 20 min, then centrifuged at 3000 g for 30 min at 4 °C. Serum was separated and stored at − 20 °C for later analysis of CORT. For gene expression, liver, spleen, epididymal white adipose tissue (EWAT), heart, and aorta were dissected from euthanized mice and stored in RNAlater solution (Thermo Fisher Scientific) at − 20 °C until RNA extraction. The brain was dissected from euthanized mice and was stored at 4 °C until RNA extraction.

### Gene expression analysis

RNA was extracted from liver, spleen, EWAT, as well as the pre-frontal cortex, hippocampus, and hypothalamus from the brain^8^ using a RNeasy mini kit (Qiagen). RNA was extracted from the heart and aorta using a RNeasy Fibrous Tissue mini kit (Qiagen). All extractions were done following the manufacturer’s instructions. RNA concentrations were quantified using a NanoDrop 2000 Spectrophotometer (ThermoScientific). cDNA was prepared by reverse transcription using a high-capacity cDNA reverse transcription kit (Life Technologies, Cat number: 4368813) according to manufacturer’s instructions.

To determine relative cytokine gene expression, RT-PCR was conducted using an ABI 7300 system. All tissues were analyzed with cytokine primers/probes (IL1β: Mm00434228, TNFα: Mm00443258, TGFβ: Mm00447500; Applied Biosystems) and heart and aorta were additionally analyzed with endothelial adhesion primers/probes (ICAM-1: Mm00516023, E-selectin: Mm00441278; Applied Biosystems). All assay probes were labeled with florescent marker 5-FAM at the 5’ end and quencher MGB a the 3’end. Primer limited 18S (4319413E; Applied Biosystems, VIC-labeled) was used as an endogenous control according to manufacturer’s instructions. Assay genes and the endogenous control were run simultaneously as a multiplex PCR assay for each sample. Samples were run in duplicate and the fold change in mRNA level was calculated as the relative mRNA expression levels (2^−ΔΔCt^)^32^. The cycle threshold (Ct) at which the fluorescence exceeded background levels was used to calculate ΔCt (Ct[target gene] – Ct[18S]). Each Ct value was normalized against the lowest Ct value of a control sample (ΔΔCt), and then the negative value of this powered to 2 (2^−ΔΔCt^) was used for analysis. Average intra- and inter-assay coefficients for variation were 0.63% and 4.70% respectfully.

### CORT measurement

Serum levels of CORT were assayed in duplicate for all animals in 5 μl following manufacturer’s protocols (ADI-900-097, Enzo Life Sciences). The kit had a sensitivity of 26.99 pg/mL and had a cross reactivity of < 30% deoxycorticosterone and < 2% progesterone. Samples were diluted 1:40 prior to running. Average intra- and inter-assay coefficients of variation were 1.56% and 3.12% respectfully. The assay conductor was blind to animal treatments.

### Statistical analysis

All analyses were conducted using RStudio (v.1.1.463, R Development Core Team, Boston, MA). A two-way ANOVA was initially used to assess the effect of drug treatment (Con, Ang, Cap), sleep fragmentation (NSF, SF), and the interaction or drug treatment and sleep fragmentation on cytokine mRNA expression and serum CORT concentrations. The interaction term was removed from the model if it was nonsignificant to preserve degrees of freedom, leaving a two-way ANOVA assessing the effect of drug treatment and sleep fragmentation on cytokine mRNA expression or serum CORT concentrations. To fit the assumptions of the ANOVA, relative gene expression was log-transformed. Tukey’s HSD post hoc test was used to parse out differences within each variable for significant results. Results are presented as mean ± SE and the significance threshold was set at *p* < 0.05.

## Results

### Peripheral response

#### EWAT

**Figure 1:**
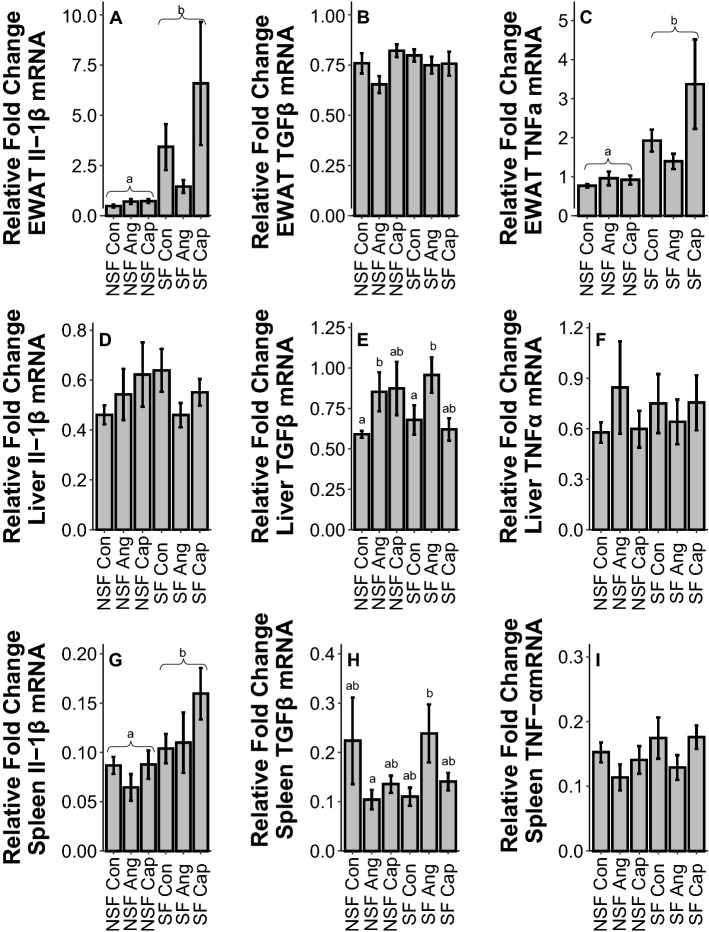
Effects of sleep fragmentation, RAS manipulation, and their interaction on Il-1β, TGFβ, and TNFα gene expression in peripheral tissues, epididymal white adipose tissue (EWAT; **A**, **B**, **C**), liver (**D**, **E**, **F**), and spleen (**G**, **H**, **I**). Samples sizes are (**A**, **B**) NSF-Con *n* = 11, NSF-Ang *n* = 10, NSF-Cap *n* = 9, SF-Con *n* = 10, SF-Ang *n* = 9, and SF-Cap *n* = 9, (**C**) NSF-Con *n* = 11, NSF-Ang *n* = 10, NSF-Cap *n* = 10, SF-Con *n* = 10, SF-Ang *n* = 9, and SF-Cap *n* = 9, (**D**) NSF-Con *n* = 10, NSF-Ang *n* = 9, NSF-Cap *n* = 10, SF-Con *n* = 10, SF-Ang *n* = 8, and SF-Cap *n* = 10, (**E**) NSF-Con *n* = 10, NSF-Ang *n* = 9, NSF-Cap *n* = 10, SF-Con *n* = 9, SF-Ang *n* = 9, and SF-Cap *n* = 9, (**F**) NSF-Con *n* = 11, NSF-Ang *n* = 9, NSF-Cap *n* = 10, SF-Con *n* = 10, SF-Ang *n* = 9, and SF-Cap *n* = 10, (**G**) NSF-Con *n* = 10, NSF-Ang *n* = 9, NSF-Cap *n* = 9, SF-Con *n* = 10, SF-Ang *n* = 9, and SF-Cap *n* = 9, (**H**) NSF-Con *n* = 10, NSF-Ang *n* = 9, NSF-Cap *n* = 8, SF-Con *n* = 10, SF-Ang *n* = 8, and SF-Cap *n* = 9, (**I**) NSF-Con *n* = 10, NSF-Ang *n* = 9, NSF-Cap *n* = 9, SF-Con *n* = 10, SF-Ang *n* = 8, and SF-Cap *n* = 9. All data were analyzed using a two-way ANOVA and Tukey’s HSD post hoc tests. Data shown as means ± 1 SE for each group and differing lowercase letters denotes *p* < 0.05.

SF increased IL1β (*F*_1,54_ = 32.73, *p* < 0.001, Fig. 1A) and TNFα (*F*_1,55_ = 23.88, *p* < 0.001, Fig. 1C) gene expression, but had no impact on TGFβ (*F*_1,54_ = 0.59, *p* = 0.45, Fig. 1B) in EWAT. Drug treatment did not alter EWAT gene expression (IL1β: *F*_2,54_ = 1.90, *p* = 0.16; TGFβ: *F*_2,54_ = 2.37, *p* = 0.10; TNFα: *F*_2,55_ = 0.90, *p* = 0.41, Fig. 1A–C).

#### Liver

SF had no effect on IL1β (*F*_1,53_ = 0.52, *p* = 0.47, Fig. 1D), TGFβ (*F*_1,52_ = 0.03, *p* = 0.87, Fig. 1E), or TNFα (*F*_1,55_ = 0.21, *p* = 0.65, Fig. 1F) gene expression in liver tissue. However, Ang increased hepatic TGFβ gene expression compared to control (*F*_2,52_ = 3.71, *p* = 0.03, Fig. 1E), but did not affect IL1β (*F*_2,53_ = 0.47, p = 0.62, Fig. 1D) or TNFα (*F*_2,55_ = 0.01, *p* = 0.99, Fig. 1F) gene expression.

#### Spleen

SF led to higher gene expression of IL1β (*F*_1,52_ = 6.47, *p* = 0.01, Fig. 1G), but not TGFβ (*F*_1,548_ = 0.38, *p* = 0.54, Fig. 1H) or TNFα (*F*_1,51_ = 1.25, *p* = 0.27, Fig. 1I). There was no effect of drug on cytokine gene expression (IL1β: *F*_2,52_ = 2.90, *p* = 0.06; TGFβ: *F*_2,48_ = 0.13, *p* = 0.88; TNFα: *F*_2,51_ = 2.01, *p* = 0.14, Fig. 1G–I). There was no significant effect of an interaction between SF and drug treatment for TGFβ gene expression in spleen (*F*_2,48_ = 2.90, *p* = 0.06, Fig. 1H).

#### Heart

**Figure 2:**
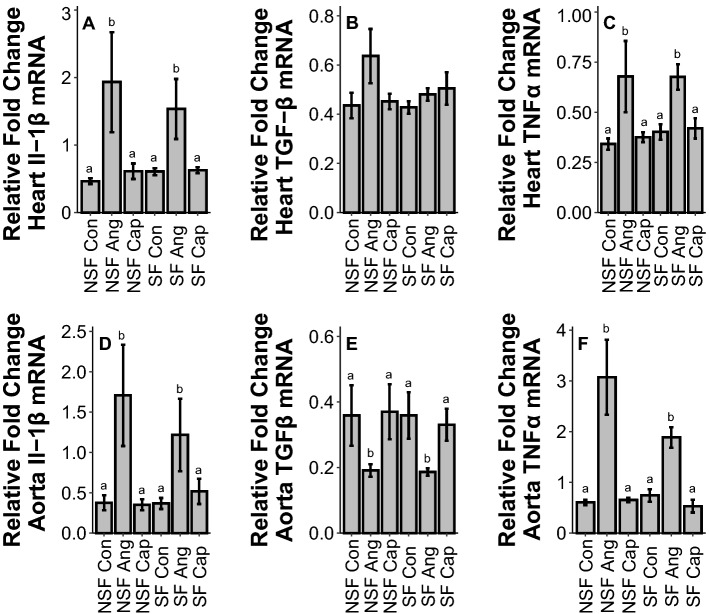
Effects of sleep fragmentation, RAS manipulation, and their interaction on Il-1β, TGFβ, and TNFα gene expression in circulatory tissues, heart (**A**, **B**, **C**) and aorta (**D**, **E**, **F**). Samples sizes are (**A**) NSF-Con *n* = 10, NSF-Ang *n* = 10, NSF-Cap *n* = 10, SF-Con *n* = 9, SF-Ang *n* = 9, and SF-Cap *n* = 10, (**B**) NSF-Con *n* = 10, NSF-Ang *n* = 10, NSF-Cap *n* = 10, SF-Con *n* = 10, SF-Ang *n* = 8, and SF-Cap *n* = 10, (**C**) NSF-Con *n* = 10, NSF-Ang *n* = 9, NSF-Cap *n* = 10, SF-Con *n* = 10, SF-Ang *n* = 9, and SF-Cap *n* = 9, (**D**) NSF-Con *n* = 11, NSF-Ang *n* = 10, NSF-Cap *n* = 9, SF-Con *n* = 9, SF-Ang *n* = 9, and SF-Cap *n* = 10, (**E**) NSF-Con *n* = 11, NSF-Ang *n* = 10, NSF-Cap *n* = 10, SF-Con *n* = 9, SF-Ang *n* = 8, and SF-Cap *n* = 10, (**F**) NSF-Con *n* = 11, NSF-Ang *n* = 10, NSF-Cap *n* = 9, SF-Con *n* = 9, SF-Ang *n* = 9, and SF-Cap *n* = 10. All data were analyzed using a two-way ANOVA and Tukey’s HSD post hoc tests. Data shown as means ± 1 SE for each group and differing lowercase letters denotes *p* < 0.05.

**Figure 3:**
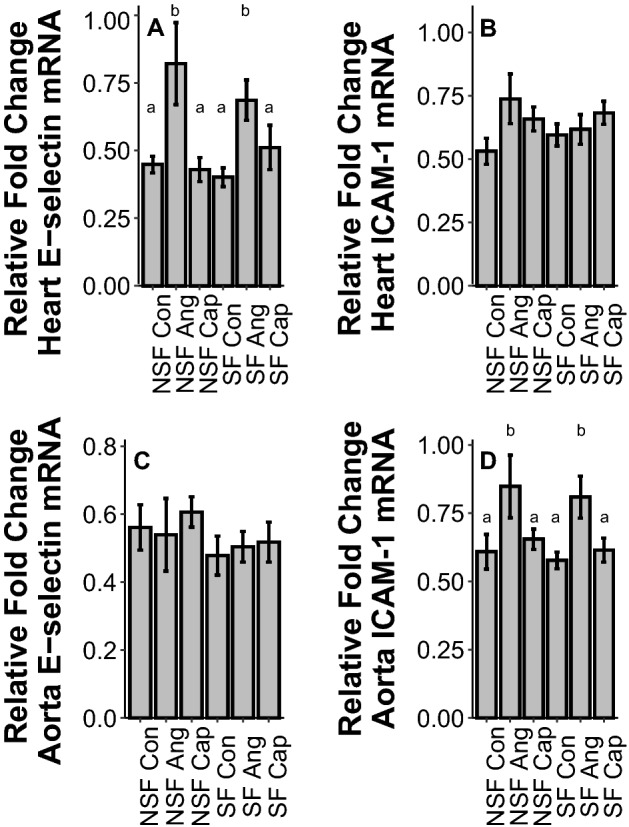
Effects of sleep fragmentation, RAS manipulation, and their interaction on E-selectin and ICAM-1 gene expression in circulatory tissues, heart (**A**, **B**) and aorta (**C**, **D**). Samples sizes are (**A**) NSF-Con *n* = 10, NSF-Ang *n* = 10, NSF-Cap *n* = 10, SF-Con *n* = 9, SF-Ang *n* = 9, and SF-Cap *n* = 10, (**B**) NSF-Con *n* = 10, NSF-Ang *n* = 10, NSF-Cap *n* = 10, SF-Con *n* = 10, SF-Ang *n* = 8, and SF-Cap *n* = 10, (**C**) NSF-Con *n* = 11, NSF-Ang *n* = 10, NSF-Cap *n* = 10, SF-Con *n* = 9, SF-Ang *n* = 9, and SF-Cap *n* = 9, (**D**) NSF-Con *n* = 11, NSF-Ang *n* = 9, NSF-Cap *n* = 10, SF-Con *n* = 9, SF-Ang *n* = 9, and SF-Cap *n* = 10. All data were analyzed using a two-way ANOVA and Tukey’s HSD post hoc tests. Data shown as means ± 1 SE for each group and differing lowercase letters denotes *p* < 0.05.

In heart, SF had no effect on cytokine or adhesion protein gene expression (IL1β: *F*_1,54_ = 1.33, *p* = 0.25; TGFβ: *F*_1,54_ = 0.07, *p* = 0.80; TNFα: *F*_1,53_ = 2.09, *p* = 0.15; E-selectin: *F*_1,54_ = 0.10, *p* = 0.75; ICAM-1: *F*_1,54_ = 0.02, *p* = 0.90, Fig. 2A–C, Fig. 3A, B). However, Ang increased IL1β (*F*_2,54_ = 7.84, p = 0.001), Fig. 2A, TNFα (F_2,53_ = 10.99, *p* < 0.001, Fig. 2C), and E-selectin gene expression (*F*_2,54_ = 9.69, *p* < 0.001, Fig. 3A) but had no impact on TGFβ (*F*_2,54_ = 2.24, *p* = 0.12, Fig. 2B) or ICAM-1 (*F*_2,54_ = 2.37, *p* = 0.10, Fig. 3B) gene expression.

#### Aorta

There was no effect of SF on gene expression in aorta (IL1β: *F*_1,54_ = 0.00, *p* = 0.98; TGFβ: *F*_1,54_ = 0.09, *p* = 0.77; TNFα: *F*_1,54_ = 2.43, *p* = 0.13; E-selectin: *F*_1,53_ = 1.58, *p* = 0.21; ICAM-1: *F*_1,54_ = 0.48, *p* = 0.49, Fig. 2D–F, Fig. 3A, B). In aorta, drug treatment significantly altered cytokine and adhesion protein gene expression: Ang increased IL1β (*F*_2,54_ = 8.26, *p* < 0.001, Fig. 2D), TNFα (*F*_2,54_ = 52.36, *p* < 0.001, Fig. 2F), and ICAM-1 (*F*_2,54_ = 7.24, *p* = 0.002, Fig. 3D), decreased TGFβ (*F*_2,54_ = 4.60, *p* = 0.01, Fig. 2E), and had no impact on E-selectin (*F*_2,53_ = 0.24, *p* = 0.79, Fig. 3C).

### Brain

#### Pre-Frontal cortex

**Figure 4:**
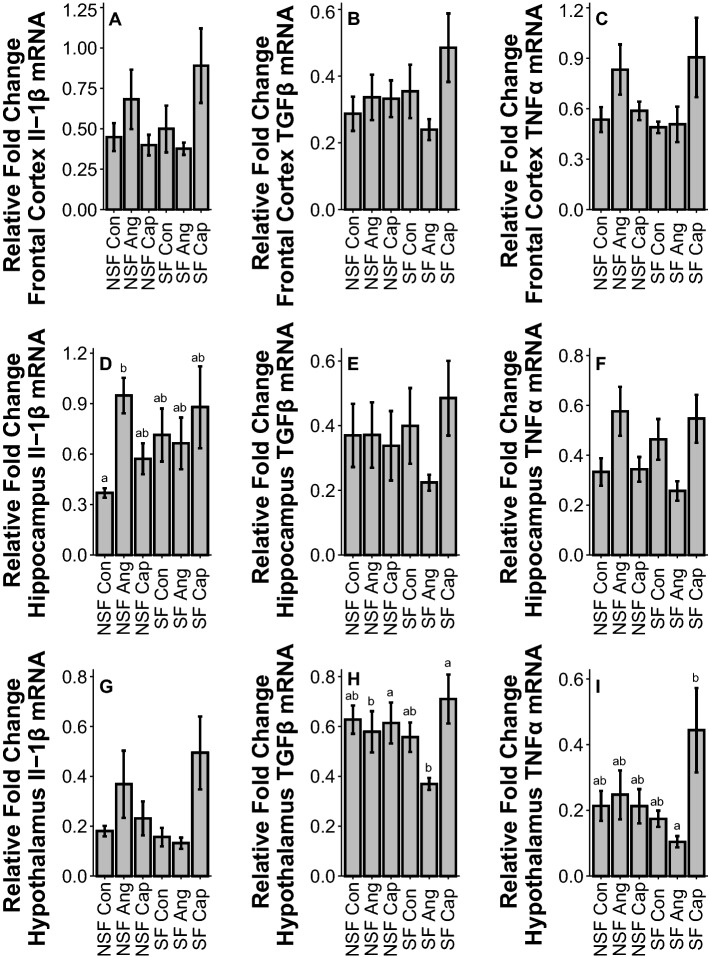
Effects of sleep fragmentation, RAS manipulation, and their interaction on Il-1β, TGFβ, and TNFα gene expression in brain tissues, prefrontal cortex (**A**, **B**, **C**), hippocampus (**D**, **E**, **F**), and hypothalamus (**G**, **H**, **I**). Samples sizes are (**A**) NSF-Con *n* = 11, NSF-Ang *n* = 10, NSF-Cap *n* = 10, SF-Con *n* = 10, SF-Ang *n* = 8, and SF-Cap *n* = 10, (**B**) NSF-Con *n* = 10, NSF-Ang *n* = 10, NSF-Cap *n* = 10, SF-Con *n* = 10, SF-Ang *n* = 8, and SF-Cap *n* = 10, (**C**) NSF-Con *n* = 11, NSF-Ang *n* = 10, NSF-Cap *n* = 10, SF-Con *n* = 9, SF-Ang *n* = 9, and SF-Cap *n* = 10, (**D**) NSF-Con *n* = 10, NSF-Ang *n* = 9, NSF-Cap *n* = 9, SF-Con *n* = 10, SF-Ang *n* = 9, and SF-Cap *n* = 10, (**E**) NSF-Con *n* = 11, NSF-Ang *n* = 10, NSF-Cap *n* = 10, SF-Con *n* = 10, SF-Ang *n* = 8, and SF-Cap *n* = 10, (**F**) NSF-Con *n* = 10, NSF-Ang *n* = 10, NSF-Cap *n* = 10, SF-Con *n* = 10, SF-Ang *n* = 8, and SF-Cap *n* = 10, (**G**) NSF-Con *n* = 10, NSF-Ang *n* = 10, NSF-Cap *n* = 10, SF-Con *n* = 9, SF-Ang *n* = 8, and SF-Cap *n* = 10, (**H**) NSF-Con *n* = 11, NSF-Ang *n* = 10, NSF-Cap *n* = 9, SF-Con *n* = 10, SF-Ang *n* = 8, and SF-Cap *n* = 10, (**I**) NSF-Con *n* = 10, NSF-Ang *n* = 9, NSF-Cap *n* = 10, SF-Con *n* = 9, SF-Ang *n* = 8, and SF-Cap *n* = 10. All data were analyzed using a two-way ANOVA and Tukey’s HSD post hoc tests. Data shown as means ± 1 SE for each group and differing lowercase letters denotes *p* < 0.05.

There was no effect of SF on cytokine gene expression in pre-frontal cortex (IL1β: *F*_2,55_ = 0.47, *p* = 0.63; TGFβ: *F*_2,54_ = 1.42, *p* = 0.25; TNFα: *F*_2,54_ = 1.71, *p* = 0.19, Fig. 4A–C) or drug treatment (IL1β: *F*_2,55_ = 0.47, *p* = 0.63; TGFβ: *F*_2,54_ = 1.42, *p* = 0.25; TNFα: *F*_2,54_ = 1.71, *p* = 0.19, Fig. 4A–C).

#### Hippocampus

There was no effect of SF on cytokine gene expression in hippocampus (IL1β: *F*_1,51_ = 0.68, *p* = 0.41; TGFβ: *F*_1,54_ = 0.21, *p* = 0.65; TNFα: *F*_1,52_ = 0.08, *p* = 0.78, Fig. 4D–F). Drug treatment had no effect on TGFβ (*F*_2,54_ = 0.36, *p* = 0.70, Fig. 4E) or TNFα (*F*_2,52_ = 0.22, *p* = 0.81, Fig. 4F) gene expression. There was an interactive effect of SF and drug treatment on IL1β (*F*_2,51_ = 4.51, *p* = 0.01, Fig. 4B) gene expression, with Ang NSF being higher than control NSF in hippocampus. Additionally, there was an interaction between sleep fragmentation and drug treatment on TGFβ (*F*_2,54_ = 6.72, *p* = 0.01, Fig. 4E); however, post hoc tests revealed no significant differences (*p* > *0.05)*.

#### Hypothalamus

There was no effect of SF on inflammatory gene expression in hypothalamus (IL1β: *F*_1,51_ = 0.25, *p* = 0.62; TGFβ: *F*_1,54_ = 0.29, *p* = 0.59; TNFα: *F*_1,50_ = 1.88, *p* = 0.18, Fig. 4G-I). Drug treatment significantly altered TGFβ (*F*_2,54_ = 3.68, *p* = 0.03, Fig. 4H) gene expression, with Cap leading to higher expression than Ang, but had no effect on IL1β (*F*_2,51_ = 1.32, *p* = 0.28, Fig. 4G) or TNFα (*F*_2,50_ = 2.31, *p* = 0.11, Fig. 4I) gene expression. There was a significant interaction of sleep treatment and drug treatment on IL1β (*F*_2,51_ = 3.61, *p* = 0.03, Fig. 4G) and TNFα (*F*_2,50_ = 4.31, *p* = 0.019, Fig. 4I) gene expression. While post hoc tests showed no differences in IL1β gene expression, TNFα gene expression was significantly higher in Cap SF compared to Ang SF (*p* < *0.05).*

### Circulating CORT

**Figure 5:**
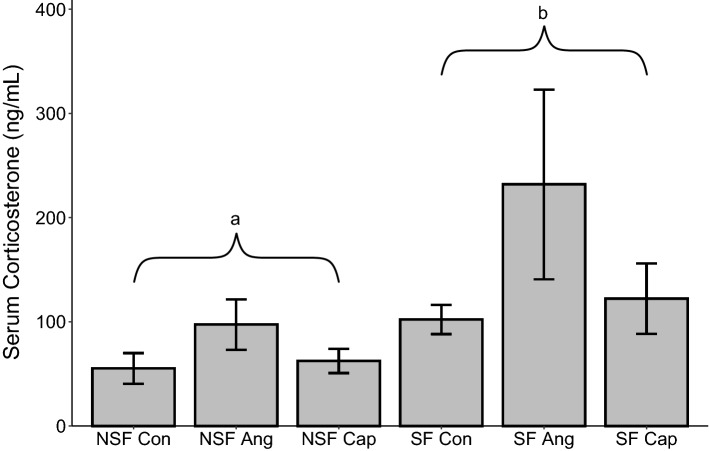
Effects of sleep fragmentation, RAS manipulation, and their interaction on plasma corticosterone levels. Samples sizes are NSF-Con *n* = 11, NSF-Ang *n* = 8, NSF-Cap *n* = 10, SF-Con *n* = 8, SF-Ang *n* = 8, and SF-Cap *n* = 10 and were analyzed using a two-way ANOVA and Tukey’s HSD post hoc tests. Data shown as means ± 1 SE for each group and lowercase differing letters denotes *p* < 0.05.

Sleep fragmentation increased serum CORT (*F*_1,51_ = 7.74, *p* = 0.01, Fig. 5) while drug treatment had no impact on serum CORT (*F*_2,51_ = 1.96, *p* = 0.15, Fig. 5).

## Discussion

These results suggest that pharmacological manipulation of the RAS altered cytokine gene expression largely independent of sleep fragmentation. Therefore, the hypothesis that alterations in Ang II affects inflammatory responses in the same way as sleep fragmentation is not supported. By extension, despite an elevation in blood pressure from angiotensin treatment, there was no overlap in the type of inflammatory responses induced by sleep fragmentation versus angiotensin treatment, suggesting that elevated blood pressure is not a major factor mediating inflammatory responses to sleep fragmentation. Therefore, the vascular stress hypothesis is not supported. In peripheral tissues, sleep fragmentation elevated cytokines in adipose and spleen tissues while Ang increased TGFβ in the liver. In heart and aorta, there was no impact of sleep fragmentation, but Ang administration increased pro-inflammatory cytokines as well as tissue-specific effects in adhesion protein genes and TGFβ. In brain tissue, Cap administration increased TGFβ in hypothalamic tissue. Additionally, we report interactive effects of RAS manipulation and sleep fragmentation in the hippocampus and hypothalamus, with Ang elevating Il-1β in NSF hippocampus and Cap elevating TNFα in SF hypothalamus. Finally, sleep fragmentation, but not RAS treatment, elevated CORT concentration in serum. Therefore, these data suggest that the pro-inflammatory effects of RAS manipulation are tissue-specific and likely differ from effects of sleep fragmentation. However, to better understand these interactive effects, additional studies need to be conducted to understand how sleep fragmentation affects RAS (and vice versa) using appropriate biomarkers (e.g., plasma angiotensin, catecholamines using microdialysis).

In peripheral tissue, sleep fragmentation and RAS treatment altered cytokines. The most responsive tissue was EWAT, showing elevations in Il-1β and TNFα gene expression due to sleep fragmentation. These results are consistent with previous work^8,33^, highlighting the important role of EWAT in the inflammatory response^34^. Liver tissue showed a marked increase in the anti-inflammatory cytokine TGFβ due to Ang administration. This elevation due to Ang treatment suggests a role of blood pressure in the development of the obesity-related liver syndrome, non-alcoholic fatty liver disease^35^. While not causally linked to liver syndromes, our data in combination with previous research, highlights a potential role of the RAS in liver dysfunction. Finally, sleep fragmentation elevated Il-1β gene expression in spleen whereas there was a trend (*p* = 0.06) for the effect of sleep fragmentation on splenic TGFβ expression to be modulated by RAS manipulation. Elevations in spleen Il-1β expression have been shown before due to sleep fragmentation^8,15^, and may be related to elevated sympathetic tone and CORT response due to stressors^36^. Previous studies have shown that acute sleep fragmentation can increase anti-inflammatory cytokines, but this effect is largely observed in the brain^8^, suggesting the spleen may be more responsive or sensitive to inflammation.

Sleep fragmentation had no effect upon cytokines or adhesion protein gene expression in the heart or aorta. Previous work using female C57BL/6j mice have reported elevated cardiac IL-1 expression from acute sleep fragmentation^15^ while current and past work with male C57BL/6j mice showed no effect (albeit at a higher rate of sleep fragmentation (120 arousals/h), there was an increase)^8,33^. These findings suggest a sex-specific vulnerability to cardiac inflammation in females compared with males^37^. Contrary to sleep fragmentation, Ang administration had many effects on circulatory mRNA expression. In heart and aortic tissue, Ang increased pro-inflammatory cytokine expression which is consistent with previous findings reporting that increased blood pressure is associated with cardiac inflammation^20^. However, the inflammatory responses seen here could be due to elevated blood pressure from Ang II or direct immunoregulatory effects from Ang II treatment^22,23,38^, and should thus be further explored. Additionally, there was upregulation of adhesion markers in both heart and aorta which become elevated in many different pathophysiological processes such as cardiovascular disorders and inflammatory responses^39,40^. Pro-inflammatory cytokines such as IL-1 and TNFα are involved with the upregulation of adhesion markers, potentially driving the elevation in adhesion markers^41,42^. The tissue-specific nature of these elevations (E-selectin in heart vs I-CAM-1 in aorta) suggests differential responses between tissue types, but further study is warranted.

Regions assessed in the brain (hypothalamus, hippocampus, and pre-frontal cortex) showed no increase in cytokine gene expression from acute sleep fragmentation. This has been a consistent finding from previous studies on C56BL/6 J male mice undergoing short-term (24 h) sleep fragmentation^8,33^, although neuroinflammation from short-term sleep restriction has been reported in rats^43^. The reasons for this species difference are unclear, but could be due to rats being more sensitive to the effects of sleep loss compared with mice^44^. There were effects from RAS treatment as well as interactions between sleep fragmentation and drug treatment in both the hippocampus and hypothalamus. In hippocampus, Ang administration resulted in elevations of Il-1β compared to control, however only in NSF. While this effect was potentially masked by elevated variance due to the stress or activity from sleep fragmentation or immunomodulatory effects of Ang, the elevations during NSF suggest Ang or blood pressure elevations can lead to inflammation in hippocampus. Pro-inflammatory cytokines, such as Il-1β, play an important role in neurogenesis, synaptic plasticity, and memory formation and consolidation at basal levels; however, elevated levels of these cytokines in the CNS are linked with behavioral and cognitive impairments, including depression^45^. Hippocampal inflammation, specifically elevated Il-1β, is linked with depressive behavior and can decrease neurogenesis, impeded learning, and decrease memory retention^46^. Nonetheless, the lack of an effect of sleep fragmentation and the interplay of sleep fragmentation with the RAS warrants further exploration as our treatments (Ang and Cap) may modulate the basic inflammatory processes involved.

For the hypothalamus, Cap administration increased the anti-inflammatory cytokine TGFβ and the pro-inflammatory cytokine TNFα, but only during sleep fragmentation. The increase in TGFβ may be a compensatory mechanism due to a decrease in blood pressure, as TGFβ administration in the hypothalamus has been shown to increase blood pressure^47^. While TGFβ is typically viewed as an anti-inflammatory cytokine^48^, it can also have pro-inflammatory impacts that may facilitate this increase in blood pressure^49^. However, the increase in TNFα due to Cap relative to Ang was counter to our hypothesis. The stimulation of the HPA axis that led to elevated CORT concentrations may play a role in regulating this pro-inflammatory cytokine due to CRH or AVP secretion^50^ or elevated TNFα may elicit a compensatory response to increased blood pressure^51^. Additionally, hypothalamic inflammation has been shown to induce and promote metabolic disease^52^. Whether this effect is mediated by alterations the RAS system is unknown. Overall, the conflicting results of the effect of Ang II and CAP on cytokines in the brain may serve to highlight the multi-faceted nature of cytokines^53^ or, alternatively, cytokine dysregulation caused by multiple stressors^54^. However, this interaction should be further explored to elucidate the underlying mechanism of these cytokines on cognitive function and disease progression.

We found consistent elevations of CORT due to sleep fragmentation, which aligns with our previous work^8,15,33^. This CORT response to sleep fragmentation is due to sympathetic nervous system activation and not changes in locomotor activity^15^. While glucocorticoids have been shown to promote development of hypertension through sodium retention^55^, we are unaware of any studies that describe a reciprocal relationship where RAS manipulation activates the HPA axis. Therefore, it is not surprising that Ang or Cap had no effect upon serum glucocorticoid concentration in this study.

## Conclusion

As world-wide obesity continues to increase^56^, the incidence of obstructive sleep apnea and elevated blood pressure are becoming more prevalent^57,58^, although our understanding of the interaction between these variables to regulate inflammation is poorly understood. We provide evidence for differential cytokine gene expression between mice exposed to pharmacological manipulation of the RAS and sleep fragmentation. Many of the changes in cytokine gene expression due to sleep fragmentation likely reflect sympathetic nervous system activation^15^, but are likely not due to changes in blood pressure. We also show that RAS manipulation alters both cytokine and epithelial gene expression in circulatory tissue such as the heart and aorta, highlighting a well-established role of RAS function in circulatory tissue pathology. Finally, we found interactions between RAS manipulation and sleep fragmentation in the hippocampal and hypothalamic regions of the brain which may have cognitive or psychiatric implications^45,59^. While these data may be due to immunomodulatory effects of our treatment, these data highlight potential inflammatory impacts of sleep fragmentation and renin-angiotensin modulation across a range of tissues and could lead to a novel understanding of the physiological ramifications of these prognoses. Future studies should explore the effect of chronic sleep fragmentation to better model obstructive sleep apnea, as well as assessing additional procedures for altering blood pressure to assess inflammatory responses.

## Supplementary Information

Supplementary Information.

## Data Availability

Data available from Dryad 10.6078/D1KD8Q^60^.
