# Supplementary material for: Contrasting effects of sleep fragmentation and angiotensin-II treatment upon pro-inflammatory responses of mice
Source: Sci Rep. 2022 Aug 30;12:14763. doi: 10.1038/s41598-022-19166-9 (PMC9427781; doi:10.1038/s41598-022-19166-9)
Supplement: Supplementary file 1 — Supplementary Information. [file 41598_2022_19166_MOESM1_ESM.docx]

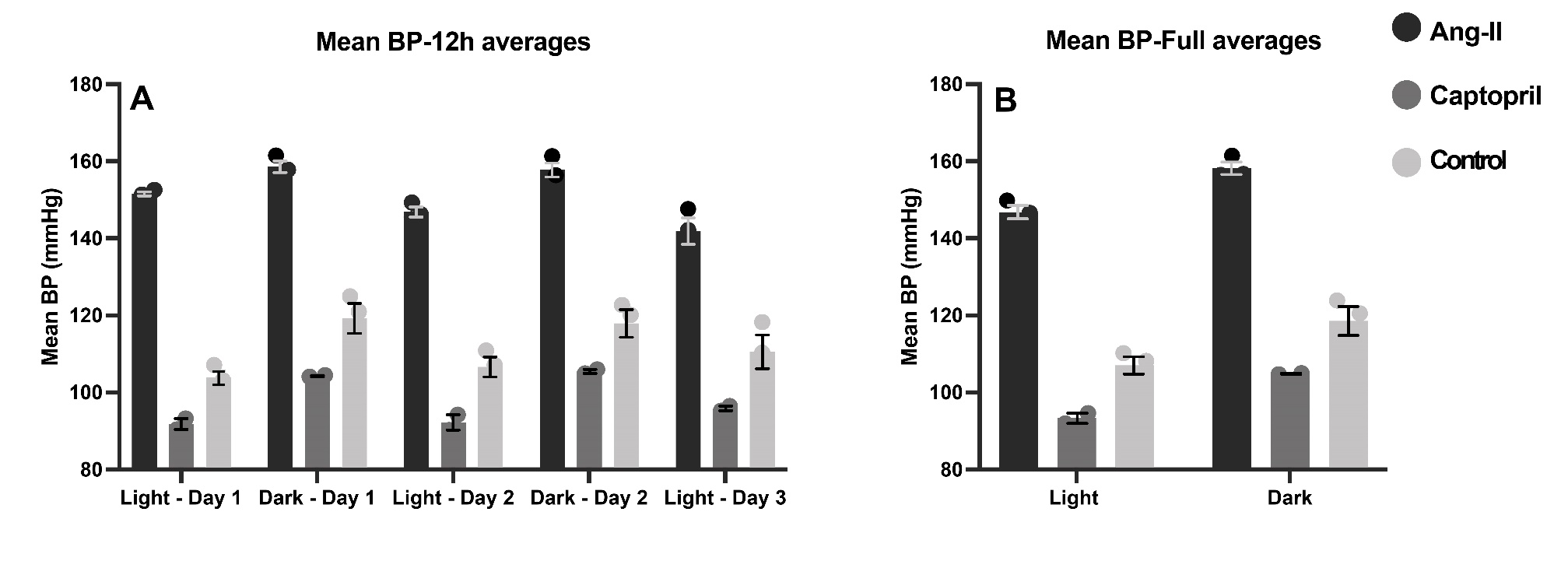


Supplementary Figure 1: Effects of Ang-II (dark grey), captopril (medium grey), and control vehicle (light grey) on mean blood pressure, measured via DSI telemetry, for daily light cycles (A) and for light vs dark averaged across days (B). Drugs were administered using osmotic pumps (Alzet model 1002) and blood pressure was measured over 48 h starting 8 days after implantation. Sample sizes are Ang-II *n*=3, captopril *n*=3, and control *n*=2. Data shown as means ± 1 SE for each group.
